# Membrane tension sensing molecule-FNBP1 is a prognostic biomarker related to immune infiltration in BRCA, LUAD and STAD

**DOI:** 10.1186/s12865-021-00475-z

**Published:** 2022-01-08

**Authors:** Zixuan Wang, Zixin Tian, Xi Song, Jun Zhang

**Affiliations:** Department of Cell Biology and Genetics, Institute of Molecular Medicine and Oncology, Chongqing Medical University, Medical School Road 1#, Yuzhong District, Chongqing, 400016 China

## Abstract

**Background:**

Formin-binding protein 1/17 (*FNBP1*/*FBP17*), as a membrane-bound protein, is wildly expressed in eukaryotic cells and performs a critical role in tumor tumorigenesis and progression. However, the relationship between *FNBP1* and immune infiltrating cells, prognostic value in patients still require comprehensive understanding. We purposed to explore the correlations of *FNBP1* expression, prognosis and immune infiltration levels in various cancers.

**Method:**

The expression and survival data of *FNBP1* were collected from Oncomine, TIMER, GEPIA, Kaplan–Meier Plotter and PrognoScan databases. Correlations between *FNBP1* and immune infiltrates were analyzed in TIMER and GEPIA databases.

**Results:**

Compared with normal tissues, *FNBP1* is significantly differentially expressed in a variety of tumor tissues. *FNBP1* has significant and complex effects on the prognosis of kinds of cancers. High-expression was obviously correlated with better prognosis in breast carcinoma and lung adenocarcinoma, while worse prognosis in stomach adenocarcinoma. Besides, FNBP1 had a correlation with various immune infiltrating cells and diverse immune gene markers in breast invasive carcinoma (BRCA), lung adenocarcinoma (LUAD), and stomach adenocarcinoma (STAD). FNBP1 was also positively correlated with the adjustment of CD8+ cells, T cells, M2 macrophage, neutrophils, monocyte, Th1 cells, T regulatory cells (Treg) and Tumor-associated macrophages (TAMs). The expression level of FNBP1 is closely positively correlated with the expression level of multiple immune checkpoints in the three cancers. In addition, FNBP1 is significantly positively correlated with the expression levels of a variety of immunosuppressive molecules.

**Conclusion:**

Our findings reveal *FNBP1* can serve as a significant biomarker to influence the prognosis and the immune infiltrating levels in different cancers. The differential expression of *FNBP1* might not only contribute to the judgment of metastatic and non-metastatic tumors but also in the immune escape by upregulating the expression of immune checkpoints.

**Supplementary Information:**

The online version contains supplementary material available at 10.1186/s12865-021-00475-z.

## Introduction

Metastasis and infiltration in cancer, as the primary cause that closely affects survival and prognosis, has become a popular topic in tumor clinical and basic research [1]. Under the difference of genetic background, in the process of tumor metastasis, the cell membrane dynamic tension structure system has changed [2]. It is caused by the dynamic assembly and reorganization of the actin skeleton in the cortex [3]. The actin skeleton assembly dynamics and rearrangement process of its cortex are distinct from that of normal cells, resulting in biological behaviors related to the actin skeleton (adhesion, migration, invasion, etc.) have undergone profound changes [4–6].

*FNBP1* (Formin-binding protein 1/17), an actin skeleton-related protein, is a member of the F-Bar/EFC family. It was isolated and identified for the first time in 1996 when the formin-interacting protein was screened from the mouse limb development expression library. It is considered to be a cell cortical actin skeleton assembly and participates in the reorganization upstream process as an important new regulator [7, 8]. *FNBP1* is widely expressed in eukaryotic cells, and its subcellular location varies with tissue cell types and their forms of existence, can exist in different subcellular divisions [9]. Previous studies have shown that *FNBP1* has a distinct F-BAR family characteristic domain, which can bind to the curved membrane [9–13]. That could change alter the tension of the plasma membrane, regulate cell polarity, and induce the tubular invagination of the cell membrane to activate actin assembly. Then it will participate in endocytosis and cell migration driven by pseudopodia [13–15]. However, in tumor research, independent research on *FNBP1* is extraordinarily limited. Only few studies have focused on migration and invasion, such as the three-dimensional movement of gastric cancer cells [16], suppression of FNBP1 affected the formation of filopodia in bladder cancer [19] and breast cancer cells [37]. Therefore, in this study, we used multiple databases for joint analysis to study the role of *FNBP1* in a variety of tumors.

TME (tumor microenvironment) and immune cells' anti-effects towards the tumor cells play a vital role in tumorigenesis. In addition, the natural immune cells (macrophages, neutrophils, dendritic cells, lymphocytes, and natural killer cells, etc.) and acquired immune cells (T cells and B cells) in TME have distinct functions [17–19]. They are respectively involved in the process of promoting or inhibiting tumor growth and are of great value to the prognosis of cancer. Therefore, it is particularly necessary to explore the characteristics and mechanisms of various immune cells.

We comprehensively analyzed the expression of *FNBP1* and its correlation with the prognostic value of pan-cancer through different databases, including Oncomine, TIMER (tumor immunity estimation resource), GEPIA, PrognoScan, and Kaplan–Meier plotter. In addition, the TIMER and GEPIA (Gene Expression Profiling Interactive Analysis) databases were used to analyze the association between *FNBP1* and the degree of immune infiltration. We have observed that *FNBP1* is widely expressed in various cancers, and may affect survival time by interacting with infiltrating immune cells.

## Methods and materials

### Oncomine database analysis

The expression level of the *FNBP1* gene in all kinds of tumors was analyzed via the Oncomine database (https://www.oncomine.org). The threshold was determined as previous studies: *P* value of 0.001, fold change of 1.5, and gene ranking of top 10% [20–22].

### PrognoScan database analysis

PrognoScan (http://dna00.bio.kyutech.ac.jp/PrognoScan/) is a powerful platform that involves a huge amount of publicly available cancer microarray datasets with corresponding clinical information. PrognoScan searches for relationships between *FNBP1* expression and patient prognosis, such as overall survival (OS), disease-free survival (DFS), Distant Metastasis Free Survival (DMFS), Disease Specific Survival (DSS), Relapse Free Survival (RFS) and so on. The threshold was adjusted to a Cox *P* value < 0.05.

### Kaplan–Meier plotter database analysis

Kaplan–Meier plotter was used for analyzing the association of *FNBP1* expression with prognosis in 7830 breast, 2190 ovarian, 3452 lung, 1440 gastric cancer patients (https://kmplot.com/analysis/) [23]. The number of patients at risk at certain time points between subgroups based on gene expression status is provided in Kaplan–Meier survival plots. The hazard ratio (HRs), 95% confidence intervals (CIs) and log-rank *P* values were calculated. A *P* value < 0.05 was considered statistically significant [24].

### Timer database analysis

Tumor Immune Estimation Resource (TIMER) is an exhaustive resource database for researching the infiltration of immune cells in tumor tissues according to RNA sequencing data from kinds of tumors (https://cistrome.shinyapps.io/timer/) [25, 26]. The Routine analysis process is as Feng and Wei’s description [27]. The study of *FNBP1* was performed by Diff Exp module, Gene module, Correlation module and Immune module. Gene markers were selected from the CellMarker database (http://biocc.hrbmu.edu.cn/CellMarker/) [28].

### Gene correlation analysis in GEPIA

Gene Expression Profiling Interactive Analysis (GEPIA) is a powerful web server for analyzing and visualizing RNA sequencing expression data [29]. Based on data from TCGA and Genotype-Tissue Expression (GTEx) Project, gene correlation was confirmed by the analysis in TIMER. The survival plots of 33 pan-cancers were analyzed by GEPIA. Correlation analysis was used on tumor and normal tissues through TCGA and GTEx datasets.

### Enrichment analysis of Gene Ontology and KEGG pathways

Gene Ontology (GO) and KEGG pathways analysis were performed by DAVID online software (https://david.ncifcrf.gov/home.jsp) [30, 31]. R software 4.0.5 (https://www.r-project.org/). were used for visualization. GO and KEGG enrichment analysis was performed as standard protocol [32–34].

### Statistical analysis

The analysis methods were performed as described [27]. Put it simply, results generated in Oncomine are shown with *P* -values determined in t-tests, fold changes, and gene ranks. The survival curve was estimated using Kaplan–Meier method. The correlation of gene expression was measured by Spearman’s correlation and statistical significance, and the degree was determined by the absolute value: 0.00–0.19 “very weak”, 0.20–0.39 “weak”, 0.40–0.59 “moderate”, 0.60–0.79 “strong”, 0.80–1.0 “very strong”. Quantitative data was shown as mean ± standard deviation. *P* < 0.05 was recognized statistically significant.

## Results

### FNBP1 expression level in diverse cancers

**Fig. 1:**
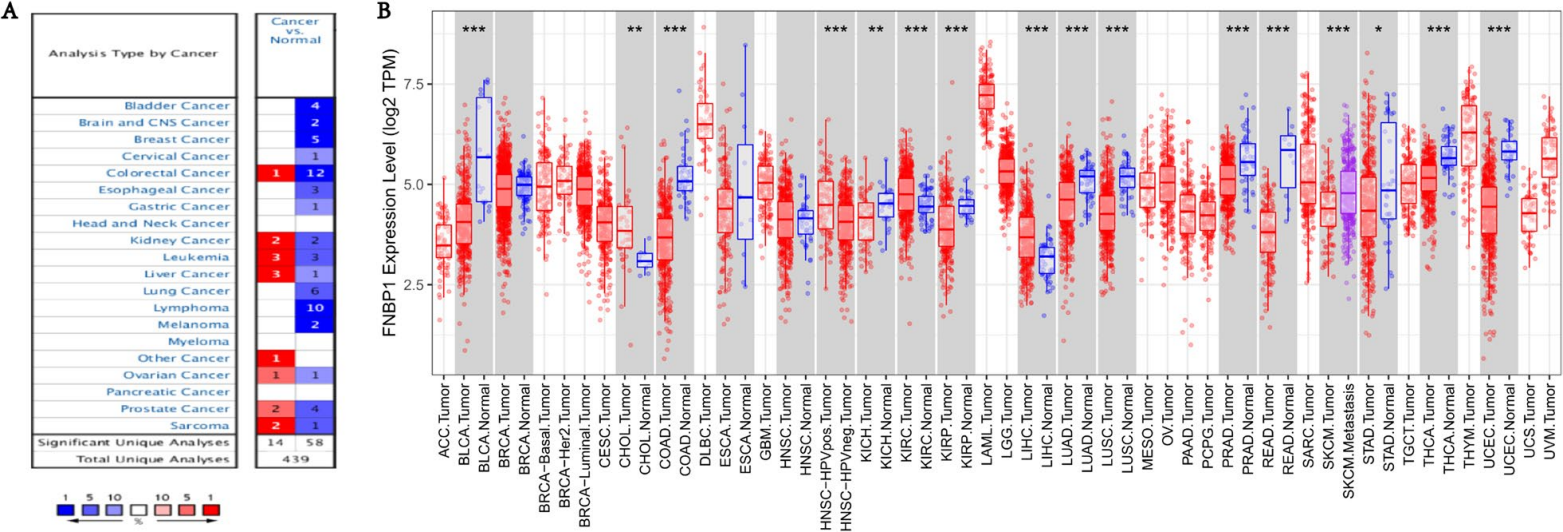
Expression of FNBP1 in various human tumors. **A** Increased or decreased expression of FNBP1 in different tumors compared to normal tissues in the Oncomine database. **B** FNBP1 expression of different tumor types from the TCGA database was explored by TIMER (*P < 0.05, **P < 0.01, ***P < 0.001)

Analyzing FNBP1 mRNA levels in various tumors and normal samples with the Oncomine database, among various cancer types, FNBP1 is significantly under-expressed in most cancer sample data sets (Fig. 1A). In addition, higher expression was found in kidney cancer, leukemia, liver cancer, ovarian cancer, prostate cancer, sarcoma and other cancer samples than in the corresponding normal samples. The specific data of FNBP1 mRNA expression levels in various cancer datasets are displayed in Additional file 2: Table S2. Consequently, we performed FNBP1 expression in multiple human cancers microarray RNA-seq data from The Cancer Genome Atlas (TCGA). Matched expression levels of FNBP1 between tumor and normal samples in all TCGA datasets are shown in Fig. 1B. In conclusion, the analysis confirmed that the expression of FNBP1 gene in cancer has changed significantly compared with normal samples.

### Prognostic value of FNBP1 in cancers

**Fig. 2:**
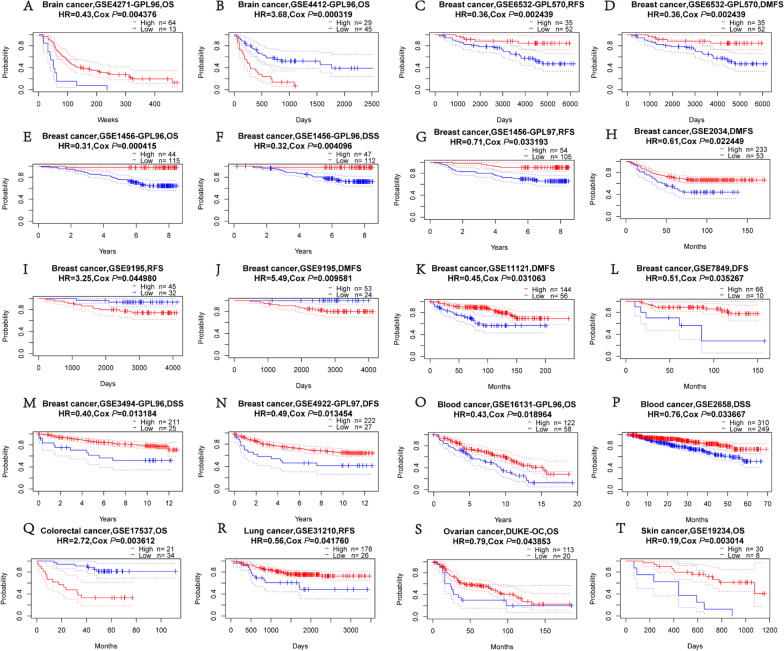
Survival Curves of high and low expression of FNBP1 in different cancers from the PrognoScan. **A** OS (n = 77) in brain cancer cohort GSE4271-GPL96. **B** OS (n = 74) in brain cancer cohort GSE4412-GPL96. **C** RFS (n = 87) in breast cancer cohort GSE6532-GPL570. **D** DMFS (n = 87) in breast cancer cohort GSE6532-GPL570. **E** OS (n = 159) in breast cancer cohort GSE1456-GPL96. **F** DSS (n = 159) in breast cancer cohort GSE1456-GPL96. **G** RFS (n = 159) in breast cancer cohort GSE1456-GPL97. **H** DMFS (n = 286) in breast cancer cohort GSE2034. **I** RFS (n = 77) in breast cancer cohort GSE9195. **J** DMFS (n = 77) in breast cancer cohort GSE9195. **K** DMFS (n = 200) in breast cancer cohort GSE11121. **L** DFS (n = 76) in breast cancer cohort GSE7849. **M** DSS (n = 236) in breast cancer cohort GSE3494-GPL96. **N** DFS (n = 249) in breast cancer cohort GSE4922-GPL97. **O** OS (n = 180) in blood cancer cohort GSE16131-GPL96. **P** DSS (n = 559) in blood cancer cohort GSE2658. **Q** OS (n = 55) in colorectal cancer cohort GSE17537. **R** RFS (n = 204) in lung cancer cohort GSE31210. **S** OS (n = 133) in ovarian cancer cohort DUKE-OC. **T** OS (n = 38) in skin cancer cohort GSE19234. OS, overall survival; DMFS, distant metastasis-free survival; DFS, disease-free survival; RFS, relapse-free survival

To investigate the correlation between *FNBP1* expression and prognosis, we evaluated the effects of *FNBP1* expression to survival via PrognoScan. Seven out of twelve cancers presented a potential correlation between *FNBP1* and prognosis (Fig. 2A–T, Additional file 2: Table S1). Six cohorts at different stages of breast cancer and showed that high *FNBP1* expression was associated with better prognosis (RFS HR = 0.36, 95%CI = 0.18 to 0.69, Cox *P* = 0.002; DMFS HR = 0.36, 95%CI = 0.18 to 0.69, Cox *P* = 0.002;OS HR = 0.31, 95%CI = 0.16 to 0.59, Cox *P* = 0.0004; DSS HR = 0.32, 95%CI = 0.15 to 0.70, Cox *P* = 0.004; RFS HR = 0.71, 95%CI = 0.51 to 0.97, Cox *P* = 0.03; DMFS HR = 0.61, 95%CI = 0.39 to 0.93, Cox *P* = 0.02; DMFS HR = 0.45, 95%CI = 0.22 to 0.93, Cox *P* = 0.03; DFS HR = 0.51, 95%CI = 0.27 to 0.95, Cox *P* = 0.03; DSS HR = 0.40, 95%CI = 0.19 to 0.83, Cox *P* = 0.01; DFS HR = 0.49, 95%CI = 0.28 to 0.86, Cox *P* = 0.01) (Fig. 2,C-H,K-N).Another group of breast cancers with a metastatic phenotype exhibited the opposite result(RFS HR = 3.25, 95%CI = 1.03 to 10.26, Cox *P* = 0.04; DMFS HR = 5.49, 95%CI = 1.51 to 19.90, Cox *P* = 0.009) (Fig. 2I–J). However, among the other common tumors, high *FNBP1* expression was correlated with a worse ending than low *FNBP1* expression in glioma and colorectal cancer. Overall, high *FNBP1* expression indicates favorable prognosis in the most of cancers beside for some metastatic tumors.

**Fig. 3:**
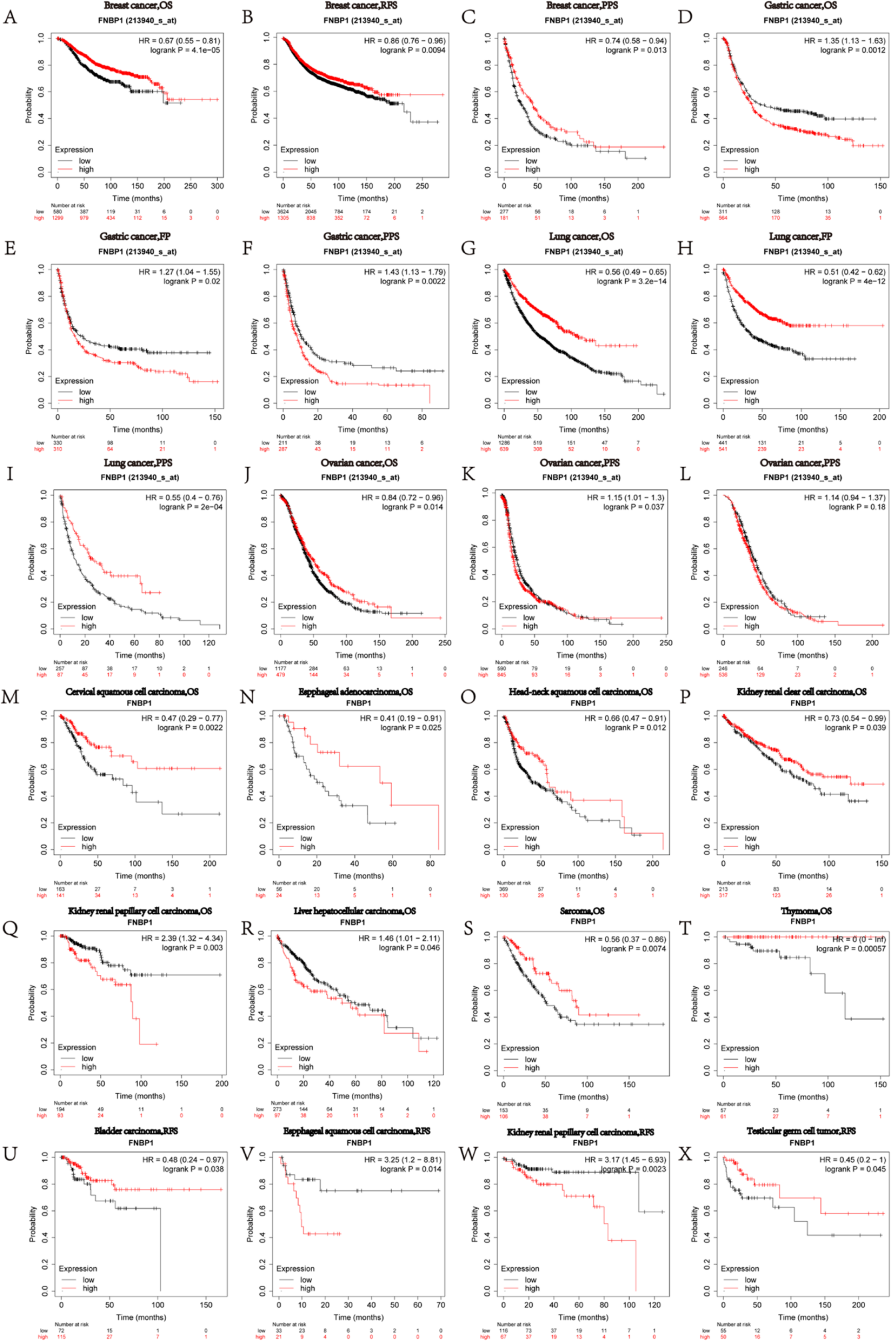
Survival Curves of high and low expression of FNBP1 in different cancers from the Kaplan–Meier Plotter. **A**, **B**, **C** OS, RFS and PPS survival curves of breast cancer (n = 1879, n = 4929 and n = 458, respectively). **D**, **E**, **F** OS, FP and PPS survival curves of gastric cancer (n = 875, n = 640 and n = 498, respectively). **G**, **H**, **I** OS, FP and PPS survival curves of lung cancer (n = 1925, n = 982 and n = 344, respectively). **J**, **K**, **L** OS, PFS and PPS survival curves of ovarian cancer (n = 1656, n = 1435 and n = 782, respectively). **M**–**T** OS survival curves of various kinds of cancers (Cervix n = 304, Esophageal n = 80, Head-neck n = 499, Kidney n = 530 and 287, Liver n = 370, Sarcoma = 159, Thymoma n = 118). (U-X) RFS survival curves of various kinds of cancers (Bladder n = 187, Esophageal n = 54, Kidney n = 183, Testicular n = 105)

Then, we further assessed the prognostic value of *FNBP1* in different tumors with Kaplan–Meier plotter, which is based on Affymetrix microarray data. Interestingly, the poor prognosis in gastric cancer(OS HR = 1.35, 95%CI = 1.13 to 1.63, *P* = 0.0012; FP HR = 1.27, 95%CI = 1.04 to 1.55, *P* = 0.02; PPS HR = 1.43, 95%CI = 1.13 to 1.79, *P* = 0.0022), ovarian cancer(PFS HR = 1.15 95CI% = 1.01 to 1.3, *P* = 0.037), kidney renal papillary cell carcinoma(OS HR = 2.39 95%CI = 1.32 to 4.34, *P* = 0.003; RFS HR = 3.17, 95%CI = 1.45 to 6.93, *P* = 0.0023), liver hepatocellular carcinoma(OS HR = 1.46, 95%CI = 1.01 to 2.11, *P* = 0.046) and esophageal squamous cell carcinoma( RFS HR = 3.25, 95%CI = 1.2 to 8.81, *P* = 0.014) was shown to correlate with higher *FNBP1* expression (Fig. 3). However, *FNBP1* expression shows a better prognosis in most cancers. These results prove the *FNBP1* expression has an important impingement on the prognosis of cancers.

Cancers included in the statistics, which relationship between *FNBP1* expression and survival is displayed in Additional file 1: Fig. S1. Comparison with FNBP1 downregulated expression, up-expression was associated with worse OS or DFS in ACC (adrenocortical carcinoma), KIRP (kidney renal papillary cell carcinoma), LGG (brain lower grade glioma), LUSC (lung squamous cell carcinoma), STAD (stomach adenocarcinoma) and UVM (uveal Melanoma). In addition, elevated FNBP1 expression was correlated with better OS or DFS in KIRC (kidney renal clear cell carcinoma), LUAD (lung adenocarcinoma) and THYM (thymoma). These results suggest the critical prognostic value of FNBP1 in certain types of cancer, demonstrating that it plays a crucial role in the progression of cancer.

### The expression level of FNBP1 is positively associated with infiltrating immune cells in breast, ovarian, lung and gastric cancers.

**Fig. 4:**
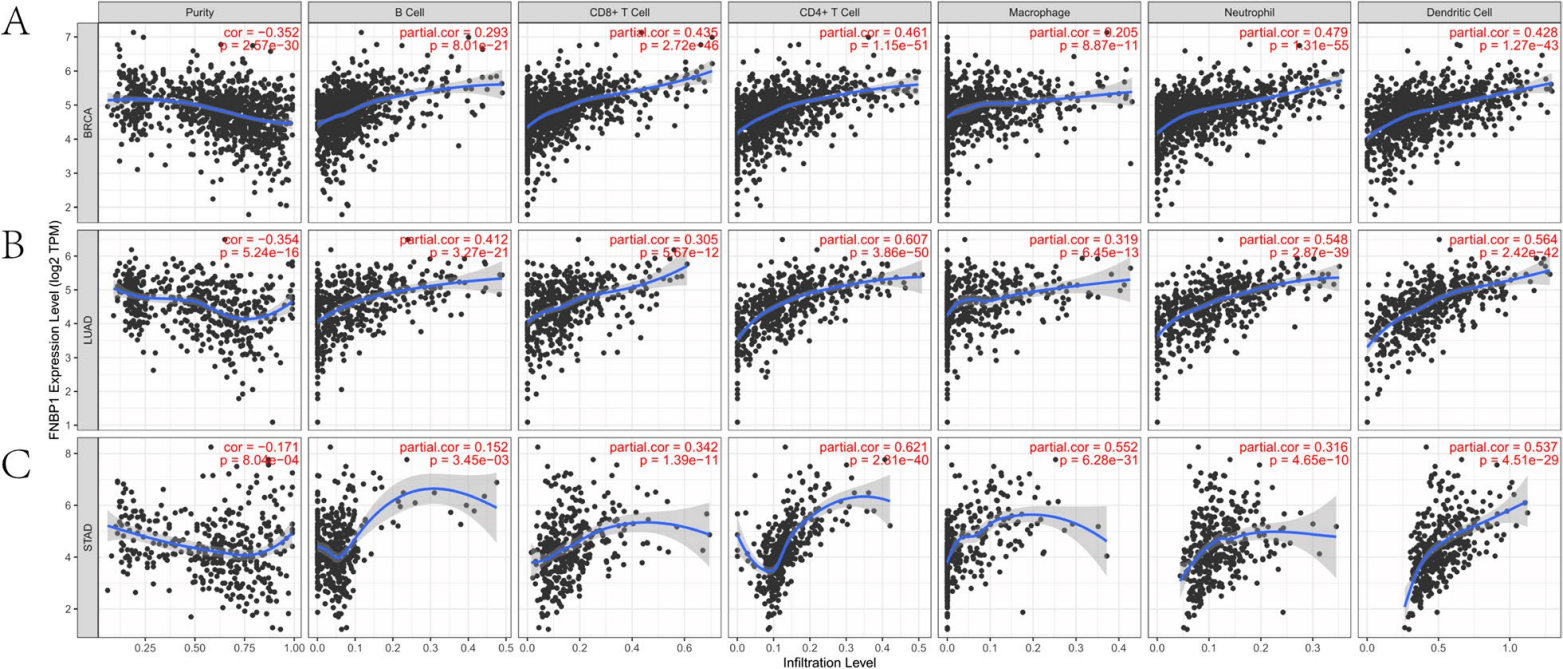
Correlation of FNBP1 expression with immune infiltration level in BRCA (breast invasive carcinoma), LUAD (lung adenocarcinoma), STAD (stomach adenocarcinoma). **A** FNBP1 expression was significantly negatively related to tumor purity and had significant positive correlations with the level of infiltrating B cells, CD8+ T cells, CD4+ T cells, macrophages, neutrophils, and dendritic cells in BRCA (n = 1093). **B** FNBP1 expression was significantly negatively related to tumor purity and had significant positive correlations with the level of infiltrating B cells, CD8+ T cells, CD4+ T cells, macrophages, neutrophils, and dendritic cells in LUAD (n = 515). **C** FNBP1 expression was negatively related to tumor purity and had significant positive correlations with the level of infiltrating B cells, CD8+ T cells, CD4+ T cells, macrophages, neutrophils, and dendritic cells in STAD (n = 415)

Immune infiltration in the tumor microenvironment is an independent predictor of survival and prognosis. Thus, the correlation between *FNBP1* and tumor-infiltrating immune cells was assessed in different cancers with TIMER. The results presented that the expression of *FNBP1* was significantly correlated with the infiltration level of B cells in 32 tumors, CD8+ T cells in 30 tumors, CD4+ T cells in 33 tumors, macrophages in 33 tumors, neutrophils in 32 tumors, and dendritic cells in 36 tumors (Additional file 1: Fig. S2). Tumor purity refers to the proportion of tumor cells in tumor tissue. Studies have shown that tumor purity is significantly related to the clinical characteristics, genome expression and biological characteristics of tumor patients. Ignoring the impact of tumor purity can lead to bias in tumor genotyping and recurrence risk [35]. Accurate assessment Tumor purity helps objectively analyze tumor samples. *FNBP1* expression in 28 types of tumors was evidently correlated with tumor purity. Moreover, *FNBP1* expression level was correlated with better prognosis and high immune infiltration in BRCA and LUAD. Expression level in *FNBP1* positive correlated with the infiltration level of B cells (BRCA: R = 0.293, *P* = 8.01E−21; LUAD:R = 0.412, *P* = 3.21E−21), CD8+ T cells (BRCA: R = 0.435, *P* = 2.72E−46; LUAD:R = 0.305, *P* = 5.67E−12), CD4+ T cells (BRCA: R = 0.461, *P* = 1.15E−51; LUAD:R = 0.607, *P* = 3.86E−50), macrophages (BRCA:R = 0.205, *P* = 8.87E−11; LUAD:R = 0.319, *P* = 6.45E−13), neutrophils (BRCA:R = 0.479, *P* = 1.31E−55; LUAD:R = 0.548, *P* = 2.87E−39), and dendritic cells (BRCA: R = 0.428, *P* = 1.27E−43; LUAD:R = 0.564, *P* = 2.42E−42). Conversely, the *FNBP1* expression level was correlated with a worse prognosis and evident immune infiltration in STAD. Expression level in *FNBP1* had positive correlation with the infiltration level of B cells (R = 0.152, *P* = 3.45E-03), CD8+ T cells (R = 0.342, *P* = 1.39E−11), CD4+ T cells (R = 0.621, *P* = 2.31E-40), macrophages (R = 0.552, *P* = 6.28E−31), neutrophils (R = 0.316, *P* = 4.65E-10), and dendritic cells (R = 0.537, *P* = 4.51E−29) (Fig. 4). These results efficiently suggested that *FNBP1* plays a precise role in immune infiltration in BRCA, LUAD and STAD.

### Correlations between clinical characteristics and FNBP1 expression in BRCA, LUAD and STAD

**Fig. 5:**
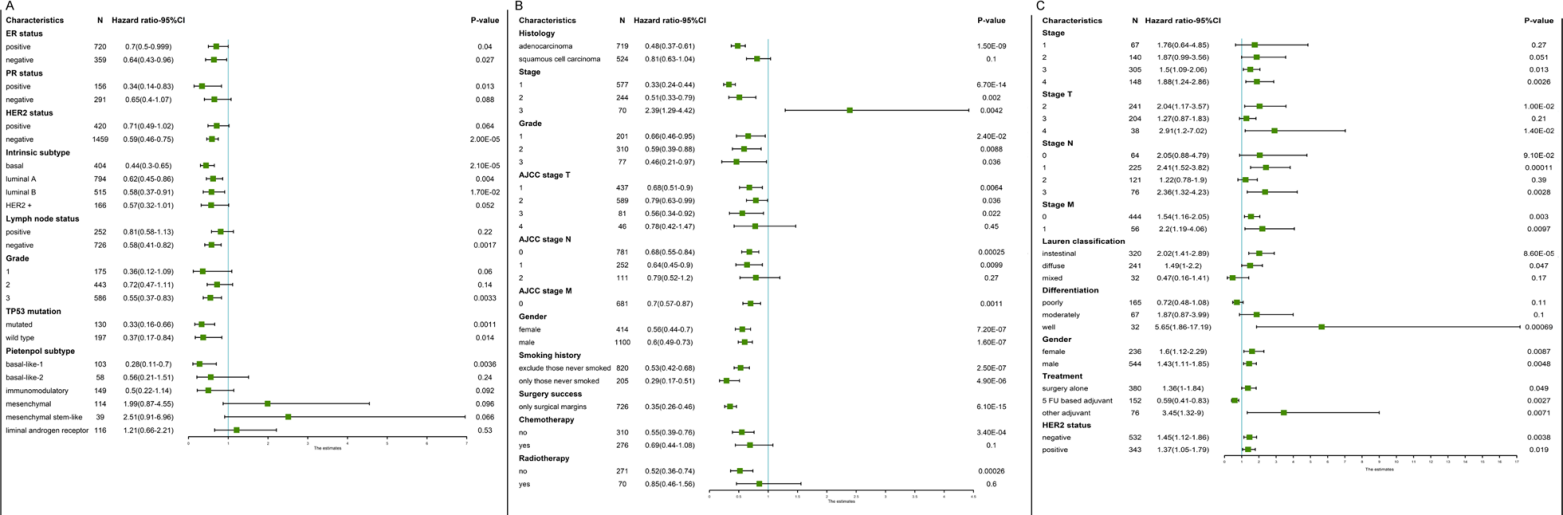
Correlation between *FNBP1* mRNA expression and prognosis in BRCA (**A**), LUAD (**B**) and STAD (**C**) with respect to clinicopathological factors

We analyzed the relationship between FNBP1 expression and clinical characteristics using R software in BRCA, LUAD and STAD patients. Better overall survival (OS) in BRCA was correlated with low FNBP1 expression (*P* < 0.05). Except for the Pietenpol subtype and low grades, low expression of FNBP1 has significant effects on various clinical factors (Fig. 5A). In LUAD patients, low FNBP1 mRNA expression was associated with worse OS in all clinical factors except stage III. In addition, FNBP1 mRNA expression had no significant results to better OS in stage T4 and N2. High expression of FNBP1 was related to better overall survival in patients who did not receive chemotherapy or radiotherapy (Fig. 5B). However, FNBP1 had contrary effects on overall survival in STAD patients. Except for patients under treatment on 5 FU based adjuvant, the overall survival rate of patients with other factors decreases with the increase of FNBP1 expression (Fig. 5C). These findings suggest that low FNBP1 mRNA expression is related to worse OS in BRCA and LUAD, and better OS in STAD. Taken together, expression of FNBP1 could be regarded as an effective prognostic indicator for breast cancers, lung adenocarcinomas and stomach adenocarcinoma depending on the clinical characteristics.

### Correlations between FNBP1 and markers of infiltrating immune cells

To demonstrate the effects of FNBP1 expression on infiltrating immune cells, we evaluated the association between FNBP1 expression and multiple markers of immune cells in BRCA, LUAD and STAD via public databases (Table 1). We calculated the correlation coefficients between FNBP1 and tumor-infiltration immune markers of the above described. Specifically, *FNBP1* expression was clearly correlated with markers of CD8+ cells, CD4+ cells, T cells, B cells, activated macrophage, neutrophils, dendritic cells, Treg cells and T cell exhaustion in LUAD, BRCA and STAD.

**Table 1:** Correlation analysis between FNBP1 and relate genes and markers of immune cells in TIMER

Description	Gene markers	BRCA (n = 1100)				LUAD (n = 515)				STAD (n = 415)			
		None		Purity		None		Purity		None		Purity	
		Cor	*P*	Cor	*P*	Cor	*P*	Cor	*P*	Cor	*P*	Cor	*P*
CD8+ T cell	CD8A	0.498	***	0.423	***	0.527	***	0.453	***	0.492	***	0.481	***
	CD8B	0.407	***	0.315	***	0.398	***	0.316	***	0.404	***	0.407	***
T cell(general)	CD2	0.513	***	0.424	***	0.594	***	0.524	***	0.512	***	0.501	***
	CD3D	0.452	***	0.349	***	0.501	***	0.405	***	0.477	***	0.461	***
	CD3E	0.503	***	0.415	***	0.608	***	0.544	***	0.507	***	0.496	***
B cell	CD19	0.355	***	0.244	***	0.416	***	0.316	***	0.552	***	0.536	***
	CD79A	0.370	***	0.259	***	0.359	***	0.257	***	0.517	***	0.487	***
Monocyte	CD115(CSF1R)	0.451	***	0.369	***	0.608	***	0.551	***	0.566	***	0.550	***
	CD86	0.444	***	0.364	***	0.557	***	0.484	***	0.440	***	0.424	***
TAM	CCL2	0.333	***	0.237	***	0.344	***	0.261	***	0.472	***	0.464	***
	CD68	0.380	***	0.301	***	0.424	***	0.356	***	0.213	***	0.206	**
	IL10	0.390	***	0.315	***	0.411	***	0.320	***	0.429	***	0.424	***
M1 Macrophage	COX2(PTGS2)	0.253	***	0.165	***	0.003	0.952	− 0.002	0.963	0.078	0.142	0.064	0.260
	INOS(NOS2)	0.110	**	0.116	**	0.176	**	0.102	0.036	− 0.037	0.489	− 0.049	0.399
	IRF5	0.252	***	0.198	***	0.488	***	0.418	***	0.338	***	0.340	***
M2 Macrophage	CD163	0.397	***	0.338	***	0.531	***	0.467	***	0.454	***	0.447	***
	MS4A4A	0.406	***	0.326	***	0.434	***	0.355	***	0.496	***	0.486	***
	VSIG4	0.300	***	0.216	***	0.389	***	0.320	***	0.410	***	0.409	***
Neutrophils	CCR7	0.470	***	0.385	***	0.559	***	0.486	***	0.632	***	0.621	***
	CD11b(ITGAM)	0.453	***	0.377	***	0.554	***	0.501	***	0.514	***	0.511	***
	CD66b(CEACAM8)	0.015	0.643	0.022	0.527	0.107	0.020	0.096	0.044	0.035	0.514	0.034	0.538
Natural killer cell	KIR2DL1	0.241	***	0.181	***	0.217	***	0.183	***	0.192	**	0.170	*
	KIR2DL3	0.278	***	0.210	***	0.287	***	0.234	***	0.134	*	0.100	0.072
	KIR2DL4	0.282	***	0.209	***	0.292	***	0.228	***	0.034	0.525	0.007	0.911
	KIR2DS4	0.240	***	0.183	***	0.282	***	0.231	***	0.152	*	0.132	0.017
	KIR3DL1	0.291	***	0.224	***	0.248	***	0.199	***	0.217	***	0.209	***
	KIR3DL2	0.332	***	0.255	***	0.323	***	0.259	***	0.302	***	0.291	***
	KIR3DL3	0.196	***	0.163	***	0.109	0.020	0.090	0.065	− 0.054	0.307	− 0.055	0.335
Dendritic cell	BDCA-1(CD1C)	0.416	***	0.311	***	0.291	***	0.218	***	0.623	***	0.622	***
	BDCA-4(NRP1)	0.376	***	0.296	***	0.341	***	0.325	***	0.573	***	0.560	***
	CD11c (ITGAX)	0.451	***	0.373	***	0.601	***	0.540	***	0.475	***	0.462	***
	HLA-DPA1	0.501	***	0.410	***	0.475	***	0.411	***	0.384	***	0.353	***
	HLA-DPB1	0.426	***	0.310	***	0.466	***	0.389	***	0.458	***	0.431	***
	HLA-DQB1	0.351	***	0.255	***	0.370	***	0.286	***	0.264	***	0.231	***
	HLA-DRA	0.521	***	0.430	***	0.425	***	0.341	***	0.352	***	0.322	***
Th1	IFN-γ (IFNG)	0.407	***	0.324	***	0.424	***	0.345	***	0.140	*	0.128	0.021
	STAT1	0.410	***	0.368	***	0.520	***	0.462	***	0.197	**	0.185	**
	STAT4	0.540	***	0.457	***	0.501	***	0.420	***	0.610	***	0.594	***
	T-bet (TBX21)	0.490	***	0.404	***	0.608	***	0.546	***	0.498	***	0.494	***
	TNF-α (TNF)	0.285	***	0.247	***	0.407	***	0.311	***	0.182	**	0.144	*
Th2	GATA3	− 0.098	*	− 0.016	0.651	0.504	***	0.442	***	0.521	***	0.508	***
	IL13	0.198	***	0.152	***	0.149	*	0.084	0.088	0.129	0.014	0.130	0.019
	STAT5A	0.332	***	0.254	***	0.709	***	0.670	***	0.512	***	0.512	***
	STAT6	0.277	***	0.274	***	0.224	***	0.255	***	0.269	***	0.283	***
Tfh	BCL6	0.215	***	0.196	***	0.301	***	0.316	***	0.601	***	0.594	***
	IL21	0.368	***	0.314	***	0.325	***	0.288	***	0.252	***	0.239	***
Th17	IL17A	0.154	***	0.088	0.010	0.199	***	0.134	*	− 0.097	0.064	− 0.102	0.067
	STAT3	0.343	***	0.334	***	0.297	***	0.327	***	0.495	***	0.493	***
Treg	CCR8	0.481	***	0.422	***	0.550	***	0.492	***	0.482	***	0.473	***
	FOXP3	0.485	***	0.407	***	0.580	***	0.520	***	0.443	***	0.426	***
	STAT5B	0.324	***	0.312	***	0.525	***	0.531	***	0.681	***	0.687	***
	TGFβ(TGFB1)	0.298	***	0.191	***	0.485	***	0.423	***	0.546	***	0.518	***
T cell exhaustion	CTLA4	0.429	***	0.331	***	0.558	***	0.480	***	0.355	***	0.339	***
	GZMB	0.369	***	0.265	***	0.366	***	0.270	***	0.099	0.060	0.056	0.331
	LAG3	0.288	***	0.212	***	0.505	***	0.428	***	0.315	***	0.301	***
	PD-1 (PDCD1)	0.394	***	0.292	***	0.529	***	0.453	***	0.419	***	0.404	***
	TIM-3(HAVCR2)	0.421	***	0.337	***	0.526	***	0.450	***	0.418	***	0.408	***

BRCA, breast invasive carcinoma; LUAD, lung adenocarcinoma; STAD stomach adenocarcinoma; TAM, tumor-associated macrophage; Th, T helper cell; Tfh, Follicular helper T cell; Treg, regulatory T cell; Cor, R value of Spearman’s correlation; None, correlation without adjustment. Purity, correlation adjusted by purity

(*P < 0.01, **P < 0.001, ***P < 0.0001)

**Fig. 6:**
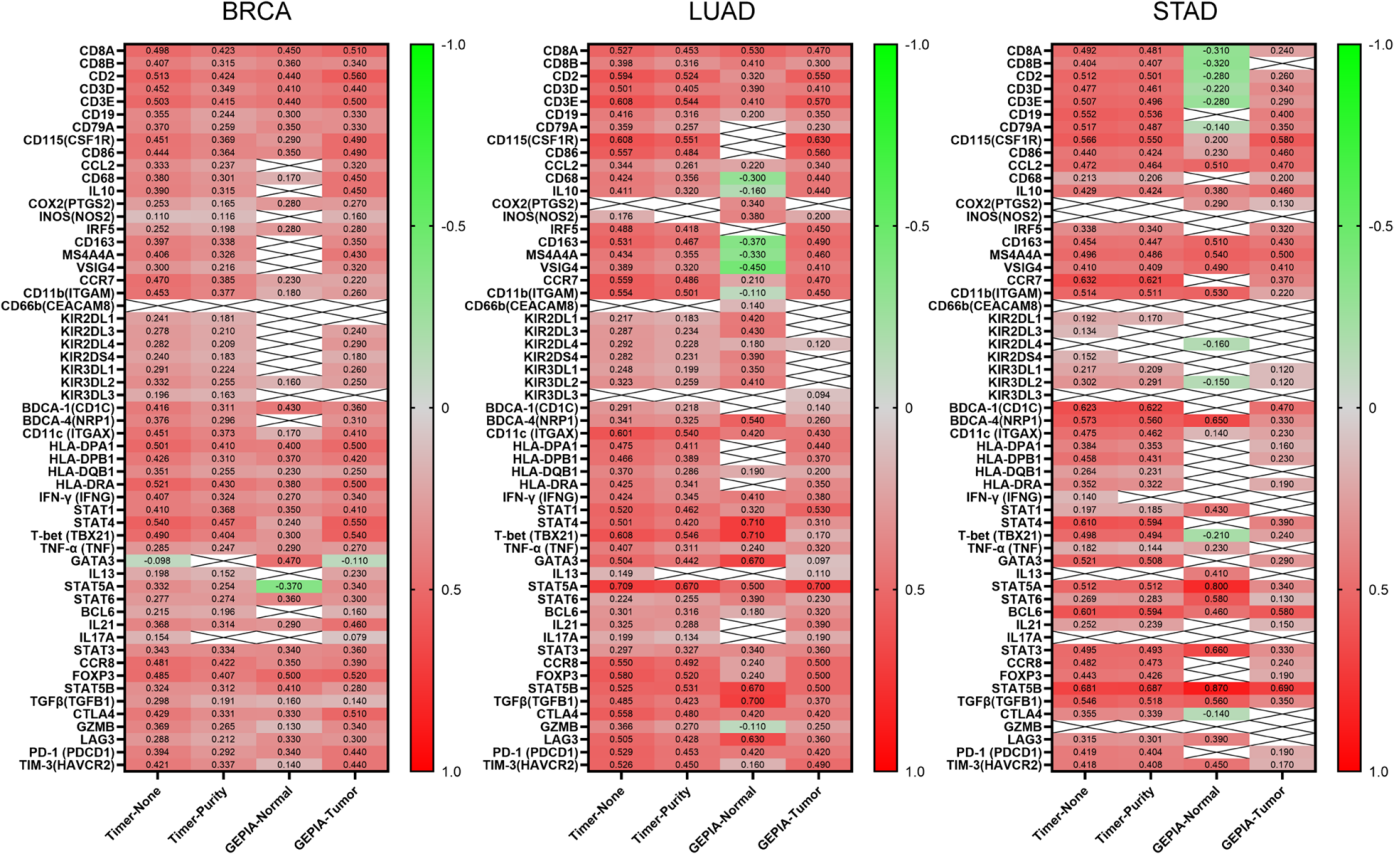
Heatmap showed FNBP1 expression correlated with immune markers in BRCA, LUAD and STAD. *Data that P greater than 0.05 is replaced by blank

We further explored the relationship between *FNBP1* and the above gene markers in matched tissues using the GEPIA database to confirm these findings (Table 2). The results showed that, in these three cancers, after tumor purity correction, FNBP1 was positively correlated in varying degrees with most immune markers. Although the results of the correlation were consistent with the TIMER analysis in tumors, from the perspective of changes in correlation strength, FNBP1 seems to play different roles in different signal regulations. These results mean that FNBP1 likely carries out multiple functions in regulating immune responses in BRCA, LUAD, and STAD, such as immune cells activation, phagocytic delivery of tumors, macrophage polarization and regulation of the T cells. (Fig. 6) Elevated FNBP1 expression levels were associated with high immune infiltration in BRCA, LUAD, and STAD. CD8+ T cell’s biomarkers (CD8A and CD8B), B cell’s biomarkers (CD19 and CD79A), Monocyte's biomarkers (CD115 and CD86), TAM's biomarkers (CCL2, CD68 and IL10), M1 macrophage’s biomarkers (COX2, NOS2, and IRF5), M2 macrophage’s biomarkers (CD163, MS4A4A, and VSIG4), neutrophil’s biomarkers (CCR7, ITGAM and CEACAM8), and dendritic cells’ biomarkers (CD1C, NRP1, ITGAX, HLA-DPA1, HLA-DPB1, HLA-DQB1, and HLA-DRA) shows high correlation with FNBP1 in BRCA, LUAD and STAD.

**Table 2:** Correlation analysis between FNBP1 and relate genes and markers of monocyte and macrophages in GEPIA

Description	Gene markers	BRCA (n = 1100)				LUAD (n = 515)				STAD (n = 415)			
		Normal		Tumor		Normal		Tumor		Normal		Tumor	
		Cor	*P*	Cor	*P*	Cor	*P*	Cor	*P*	Cor	*P*	Cor	*P*
CD8+ T cell	CD8A	0.450	***	0.51	***	0.530	***	0.470	***	− 0.31	***	0.240	***
	CD8B	0.360	***	0.34	***	0.41	***	0.300	***	− 0.32	***	0.059	0.230
T cell (general)	CD2	0.440	***	0.56	***	0.32	***	0.550	***	− 0.28	***	0.260	***
	CD3D	0.410	***	0.44	***	0.39	***	0.410	***	− 0.22	**	0.340	***
	CD3E	0.440	***	0.5	***	0.41	***	0.570	***	− 0.28	***	0.290	***
B cell	CD19	0.300	***	0.33	***	0.2	***	0.350	***	− 0.11	0.12	0.400	***
	CD79A	0.350	***	0.33	***	0.035	0.51	0.230	***	− 0.14	*	0.350	***
Monocyte	CD115(CSF1R)	0.290	***	0.490	***	− 0.010	0.860	0.630	***	0.200	*	0.580	***
	CD86	0.350	***	0.490	***	0.065	0.23	0.56	***	0.23	**	0.46	***
TAM	CCL2	0.023	0.700	0.320	***	0.22	**	0.34	***	0.51	***	0.47	***
	CD68	0.170	*	0.450	***	− 0.3	***	0.44	***	0.059	0.39	0.2	***
	IL10	0.000	1.000	0.450	***	− 0.160	*	0.440	***	0.380	***	0.460	***
M1 Macrophage	COX2(PTGS2)	0.280	***	0.270	***	0.340	***	0.014	0.750	0.290	**	0.130	*
	INOS(NOS2)	0.038	0.520	0.160	***	0.380	***	0.200	***	− 0.059	0.390	− 0.011	0.820
	IRF5	0.280	***	0.280	***	− 0.002	0.970	0.450	***	0.034	0.630	0.320	***
M2 Macrophage	CD163	− 0.130	0.026	0.350	***	− 0.370	***	0.490	***	0.510	***	0.430	***
	MS4A4A	0.056	0.340	0.430	***	− 0.33	***	0.46	***	0.54	***	0.5	***
	VSIG4	− 0.098	0.096	0.320	***	− 0.450	***	0.410	***	0.490	***	0.410	***
Neutrophils	CCR7	0.230	***	0.22	***	0.21	***	0.470	***	0.069	0.32	0.370	***
	CD11b(ITGAM)	0.180	**	0.26	***	− 0.11	*	0.450	***	0.53	***	0.220	***
	CD66b(CEACAM8)	− 0.062	0.29	− 0.018	0.55	0.14	**	− 0.021	0.65	0.068	0.33	0.011	0.83
Natural killer cell	KIR2DL1	− 0.072	0.22	0.004	0.9	0.42	***	− 0.038	0.41	− 0.003	0.97	0.041	0.41
	KIR2DL3	0.072	0.22	0.24	***	0.43	***	0.073	0.11	0.023	0.74	0.047	0.34
	KIR2DL4	− 0.025	0.67	0.29	***	0.18	***	0.120	**	− 0.16	*	− 0.065	0.19
	KIR2DS4	− 0.028	0.63	0.18	***	0.39	***	0.027	0.55	0.037	0.59	0.045	0.36
	KIR3DL1	− 0.043	0.46	0.26	***	0.35	***	0.088	0.054	0.043	0.53	0.120	*
	KIR3DL2	0.160	**	0.25	***	0.41	***	− 0.016	0.72	− 0.15	*	0.120	*
	KIR3DL3	0.010	0.86	0.043	0.16	0.052	0.34	0.094	*	− 0.11	0.1	− 0.062	0.21
Dendritic cell	BDCA-1(CD1C)	0.430	***	0.36	***	− 0.039	0.47	0.140	**	0.097	0.16	0.470	***
	BDCA-4(NRP1)	0.008	0.89	0.31	***	0.54	***	0.260	***	0.65	***	0.330	***
	CD11c (ITGAX)	0.170	**	0.41	***	0.42	***	0.430	***	0.14	*	0.230	***
	HLA-DPA1	0.400	***	0.5	***	0.058	0.28	0.440	***	− 0.027	0.7	0.160	***
	HLA-DPB1	0.370	***	0.42	***	0.06	0.26	0.370	***	0.038	0.59	0.230	***
	HLA-DQB1	0.230	***	0.25	***	0.19	***	0.200	***	− 0.094	0.17	0.048	0.34
	HLA-DRA	0.380	***	0.5	***	− 0.046	0.4	0.350	***	− 0.078	0.26	0.190	***
Th1	IFN-γ (IFNG)	0.270	***	0.34	***	0.41	***	0.380	***	− 0.048	0.48	0.035	0.48
	STAT1	0.350	***	0.41	***	0.32	***	0.530	***	0.43	***	0.052	0.3
	STAT4	0.240	***	0.55	***	0.71	***	0.310	***	− 0.07	0.31	0.390	***
	T-bet (TBX21)	0.300	***	0.54	***	0.71	***	0.17	***	− 0.21	***	0.240	***
	TNF-α (TNF)	0.290	***	0.27	***	0.24	***	0.32	***	0.23	***	− 0.010	0.84
Th2	GATA3	0.470	***	− 0.11	***	0.67	***	0.097	*	− 0.031	0.65	0.290	***
	IL13	0.110	0.069	0.23	***	0.07	0.19	0.11	*	0.41	***	0.051	0.3
	STAT5A	− 0.370	***	0.34	***	0.5	***	0.7	***	0.8	***	0.340	***
	STAT6	0.360	***	0.3	***	0.39	***	0.23	***	0.58	***	0.130	*
Tfh	BCL6	− 0.099	0.091	0.16	***	0.18	***	0.32	***	0.46	***	0.580	***
	IL21	0.290	***	0.46	***	0.074	0.17	0.39	***	− 0.09	0.19	0.150	***
Th17	IL17A	0.067	0.26	0.079	**	0.06	0.27	0.19	***	− 0.11	0.097	− 0.074	0.14
	STAT3	0.340	***	0.36	***	0.34	***	0.36	***	0.66	***	0.330	***
Treg	CCR8	0.350	***	0.39	***	0.24	***	0.5	***	− 0.1	0.14	0.240	***
	FOXP3	0.500	***	0.52	***	0.24	***	0.5	***	− 0.1	0.13	0.190	***
	STAT5B	0.410	***	0.28	***	0.67	***	0.5	***	0.87	***	0.690	***
	TGFβ(TGFB1)	0.160	**	0.14	***	0.7	***	0.37	***	0.56	***	0.350	***
T cell exhaustion	CTLA4	0.330	***	0.51	***	0.42	***	0.42	***	− 0.14	*	0.014	0.78
	GZMB	0.130	*	0.34	***	− 0.11	*	0.25	***	− 0.088	0.2	− 0.016	0.74
	LAG3	0.330	***	0.3	***	0.63	***	0.36	***	0.39	***	0.057	0.25
	PD-1 (PDCD1)	0.340	***	0.44	***	0.42	***	0.42	***	− 0.11	0.11	0.190	***
	TIM-3(HAVCR2)	0.140	*	0.44	***	0.16	**	0.49	***	0.45	***	0.170	***

BRCA, breast invasive carcinoma; LUAD, lung adenocarcinoma; STAD stomach adenocarcinoma; TAM, tumor-associated macrophage; Tumor, correlation analysis in tumor tissue of TCGA. Normal, correlation analysis in normal tissue of TCGA

(*P < 0.01, **P < 0.001, ***P < 0.0001)

### FNBP1 co-expression gene cluster promotes immune responses and potential immune escape

**Fig. 7:**
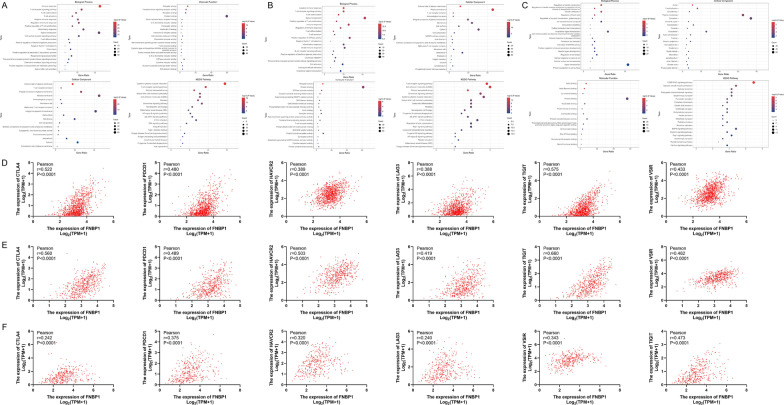
GO-KEGG and Immune Checkpoints analysis in BRCA, LUAD and STAD. Bubble graphs of GO-KEGG analysis in BRCA (**A**), LUAD (**B**) and STAD (**C**). Correlation between FNBP1 expression and immune checkpoints in BRCA (**D**), LUAD (**E**) and STAD (**F**)

We performed GO-KEGG enrichment analysis for FNBP1 co-expressed genes in BRCA, LUAD and STAD (Fig. 7A–C). We found that the FNBP1 positive-related gene group in BRCA and LUAD is highly enriched in plasma membrane function and immune-related pathways, especially in the T cell receptors' related functions. However, there is no significant enrichment of immune-related pathways in the STAD sample group, but more enriched in the basic cell biological functions such as cell junction, shape and signal transduction, etc. This result shows that FNBP1 may play a key role in tumorigenesis and development through a complex molecular network, rather than a common membrane tension sensing-actin skeleton assembly system. Detailed results of correlation analysis and GO-KEGG enrichment analysis of FNBP1 were shown in Additional file 2: Table S3-S8.

Interestingly, we also observed a significant positive correlation between FNBP1 and some important immune checkpoints (CTLA4, PDCD1, HAVCR2, LAG3, TIGIT, VSIR). Immune checkpoints often play an inhibitory function in the immune process of tumors. We noticed FNBP1 has a significant and highly positive correlation with the six immune checkpoints in the three tumors, especially in BRCA and LUAD (Fig. 7D–F). These results further confirm the correlation between FNBP1 and infiltrating immune cells in the microenvironment of BRCA, LUAD, and STAD and imply that FNBP1 participates in the process of tumor immune escape under the activation of immune responding.

## Discussion

We analyzed the mRNA expression levels of FNBP1 and the prognostic phenotype in various cancers comprehensively. Compared with normal tissues, FNBP1 expression was significantly lower in BLCA, COAD, KICH, LUAD, LUSC, PRAD, READ, STAD, THCA, and UCEC and was significantly higher in CHOL, KIRC, and LIHC. Analyses of prognostic values show that in BRCA and LUAD, besides the metastatic tumors, low levels of FNBP1 are associated with a worse prognosis. Conversely, high levels of FNBP1 are related to the poor prognosis in STAD significantly. FNBP1 expression patterns rest with the type of tumor, suggesting that FNBP1 expression can be considered as a predictive and potential marker in cancer. Not only that, we found that in BRCA, LUAD, and STAD, the expression of different immune markers and immune-infiltrating correlate with FNBP1 expression. Therefore, this study offers new visions into the immunoregulatory function of FNBP1 in BRCA, LUAD, and STAD and its application as a tumor biomarker.

Previous studies on *FNBP1* have shown high agreement on invasive tumors. The high expression of *FNBP1* is closely related to the formation of invadopodia in breast, gastric, and bladder cancer, which supports the highly invasive characteristics of tumor cells.

Hayato Yamamoto et al. proved that the membrane deformation activity mediated by *FNBP1* and the recruitment of dynamin-2 is necessary for the formation of invadopodia. *FNBP1* plays a key role in the invasion of bladder tumor cells by mediating the formation of invasive pseudopodia [36]; Prabhat Suman et al. found that breast cancer cells knocked down by *FNBP1* showed defects in the invasion phenotype, and the degradation of ECM during the invasion process was impaired, indicating that *FNBP1* is essential in the role of breast cancer cell invasion [37]. Bo Kyung Yoon et al. indicated that *FNBP1*, as the crux to high-level cell motility, exists in aggressive GC cells. The loss of *FNBP1* leads to the decrease of invasion ability, especially in the three-dimensional culture system. Sp1 motif-driven *FNBP1* expression is a key molecule process in explaining the invasiveness of EMT-type GC cells. Pharmacological inhibition and knockdown of Sp1 down-regulate *FNBP1* promoter activity and transcription level, respectively [16].

This seems to be inconsistent with the results of our data analysis, but the cancer progression associated with high *FNBP1* is mostly manifested in invasive tumor cells, such as SKCM/SKCM-metastasis group in TIMER-Pan-cancer analysis. That may be one of the molecular mechanisms by which *FNBP1* mediates this complex biological response. We believe that the main reason for this inconsistency is that our study analyzed the expression of *FNBP1* at the overall level, and did not highlight tumors with a clear invasion phenotype. At present, the research on the mechanism of *FNBP1* supporting tumor cell invasion and migration is very limited. As a membrane tension-sensing molecule, the EFC domain of *FNBP1* can form a dimer, which can sense changes in cell membrane tension in real-time, and combine with the curved plasma membrane to increase the tension, so that the migrating cells maintain polarity and make corresponding feedback adjustments. This process is a key step in cell migration. Its accumulation on the front edge of cell movement can continuously recruit N-WASP, which in turn activates the downstream Arp2/3 complex, assembles the actin branch skeleton, and forms stable, strong, and powerful filopodia, which is its powerful invasion structural basis. PrognoScan analysis based on GEO data revealed that lower FNBP1 expression associated with a poorer prognosis for diverse cancer types, such as astrocytoma, breast(non-metastasis), blood, lung, ovarian, and skin, while elevated FNBP1 expression correlated with a worse prognosis in colorectal cancer and metastatic breast cancer. In addition, Kaplan–Meier plotter analysis indicated decreased FNBP1 expression was related to short survival in breast, lung, cervical, esophageal, head-neck, kidney renal clear carcinoma, bladder, and testicular cancer patients. Depletion of FNBP1 led to the worse OS in BRAC patients with any status ER, positive PR, negative HER2, mutant and non-mutant in TP53. The knowledge that immune cells can recognize and destroy cancer cells has promoted an enormous change in the understanding of cancer, and immunotherapy has been proved to be effective for tumors that are resistant to conventional treatments [38]. Another conclusion in our research was raised that the level of FNBP1 had an association with various types of immune infiltration in tumors, especially in BRCA, LUAD, and STAD. Our results revealed that the FNBP1 expression level had a significant positive correlation with infiltration levels of most kinds of infiltrating immune cells. Interestingly, analyzing by GEPIA database, FNBP1 negatively correlated with TAM immune markers in LUAD and CD8+ T cell's immune markers in STAD in normal tissues. In the process of cancer, the correlation between FNBP1 and immune markers changes from negative to positive. This result indicates that FNBP1 also plays an important role in the polarization of tumor macrophages.

Immune escape is a central issue targeted at immunotherapy. Tumor cells would be difficult to be recognized in growth and metastasis under the high expression level of the immune checkpoints, although innate immune cells are activated. This is because immunosuppressive molecules can inhibit the activation of anti-tumor cells such as CD4+ T cells and NK cells by competitively binding to epitopes of immune cells. The expression level of FNBP1 is highly positively correlated with a variety of immune checkpoint molecules. We speculate that FNBP1 establishes a connection with cell membrane epitopes through the plasma membrane-tonicity sensing system, affecting its expression.

CTLA-4 is mainly expressed on T cells and is a negative regulatory receptor for T cells. It competes with CD28 for binding to CD80 and CD86 with higher affinity, leading to the inactivation of T cells [39]. According to existing studies, anti-CTLA-4 treatment will promote the depletion of Treg in the tumor microenvironment, indicating that CTLA-4 contributes to Tregs' activation. Immoderate Tregs could keep the immune response from killing cancer cells and promote cancer progression [40].

PDCD1 (PD-1) is an inhibitory receptor expressed by a variety of immune cells. The combination with its highly specific ligands PD-L1 and PD-L2 can deplete effector T cells and fail to recognize target cells [41–43]. TIGIT is an inhibitory receptor expressed on NK cells, CD8+ T, CD4+ T and Treg cells. After TIGIT is activated by its cognate ligand, it inhibits the activation of NK cells and CD4+ T cells [44]. In clinical models, the combined blocking of PD-L1 and TIGIT can better recover the anti-tumor immune function than blocking PD-L1 alone. In previous pre-clinical murine cancer models, co-blockade of LAG3 and PDCD1 induced an up-regulation anti-tumor response [45, 46]. LAG3 also contributes to tumor immune escape [47]. Besides, TIM-3 (HAVCR2) mediates T cell exhaustion and macrophage activation; Continuous depletion of T cells downregulates the immune response in tumor-carrying hosts [48].

The high correlation between FNBP1 and many immune checkpoints reminds us: Abnormal membrane actin skeleton assembly ability is a necessary condition for tumor cell metastasis. Therefore, the high expression of FNBP1 not only provides the motivation for tumor cell metastasis but also has a strong relationship with the potential association of immune checkpoints that prevent tumors from surveillance attacks by the immune system during metastasis.

As a summary of the above, we believed that FNBP1 plays a critical role in tumorigenesis.

In this study, there were several deficiencies. First, the cutoff values in the different online databases were inconsistent, which inevitably introduce potential heterogeneity. Second, the online samples updating was still untimely. Thus, in the next task, more numerous samples are required to provide more clear evidence to confirm the effect of FNBP1 on tumor immune infiltration. Another project we are focusing on is to explore the mechanism of FNBP1 in tumorigenesis of various cancers and its relationship with immune infiltration in animal experiments in vitro and in vivo. Therefore, further research is needed to verify the role of FNBP1 in interested cancers using these models.

This is the first study in which FNBP1 has been reported as a new biomarker for many kinds of tumors. It reveals the role of FNBP1 in immune cell infiltration. With further understanding of its functional scope, the involved mechanism of FNBP1 may become an effective tool for distinguishing and diagnosis primary and metastasis tumors.

## Supplementary Information

**Additional file 1**. Supplementary Figures.

**Additional file 2**. Supplementary Tables.

## Data Availability

The datasets used and/or analyzed in the current study are available from the corresponding author upon reasonable request. All data generated or analyzed during this study are included in this published article.
