# Supplementary material for: Membrane tension sensing molecule-FNBP1 is a prognostic biomarker related to immune infiltration in BRCA, LUAD and STAD
Source: BMC Immunol. 2022 Jan 8;23:1. doi: 10.1186/s12865-021-00475-z (PMC8742955; doi:10.1186/s12865-021-00475-z)
Supplement: Supplementary file 1 — Additional file 1. Supplementary Figures. [file 12865_2021_475_MOESM1_ESM.pdf]

Supplementary Figure 1. Correlation of FNBP1 expression with prognosis in diverse types of cancer.

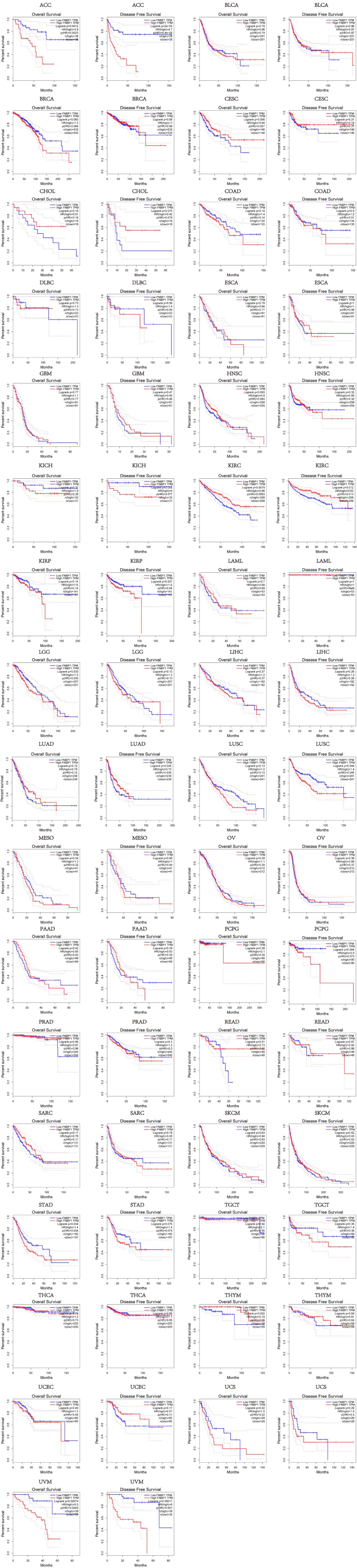

Supplementary Figure 2. Correlation of FNBP1 expression with tumor-infiltrating immune cells in various types of cancers via TIMER database.

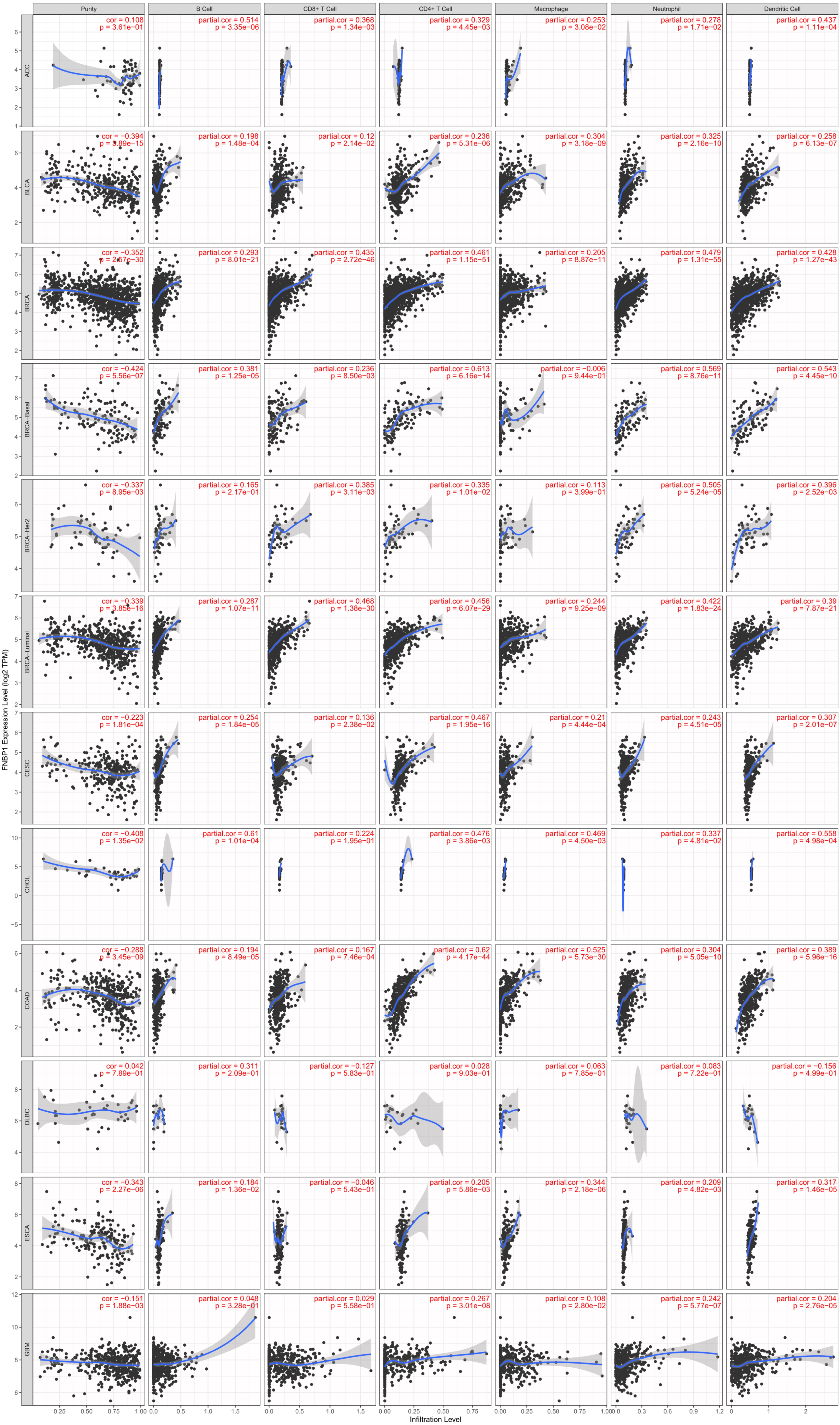

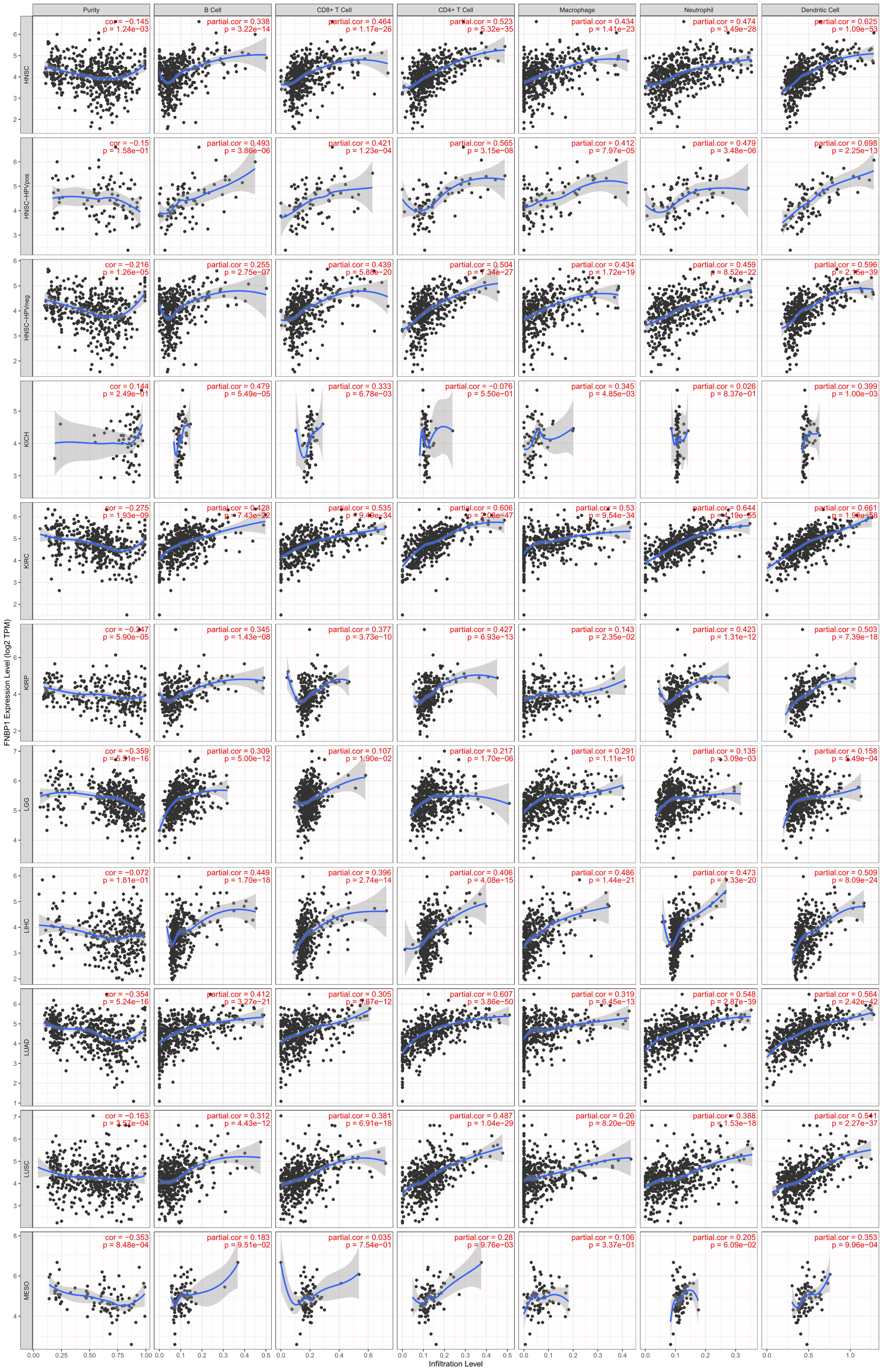

Purity
B Cell
CD8+ T Cell
CD4+ T Cell
Macrophage
Neutrophil
Dendritic Cell
HNSC
cor = −0.145 p = 1.24e−03
partial.cor = 0.338 p = 3.22e−14
partial.cor = 0.464 p = 1.17e−26
partial.cor = 0.523 p = 5.32e−35
partial.cor = 0.434 p = 1.41e−23
partial.cor = 0.474 p = 3.49e−28
partial.cor = 0.625 p = 1.09e−53
HNSC-HPVpos
cor = −0.15 p = 1.58e−01
partial.cor = 0.493 p = 3.86e−06
partial.cor = 0.421 p = 1.23e−04
partial.cor = 0.565 p = 3.15e−08
partial.cor = 0.412 p = 7.97e−05
partial.cor = 0.479 p = 3.48e−06
partial.cor = 0.698 p = 2.25e−13
HNSC-HPVneg
cor = −0.216 p = 1.26e−05
partial.cor = 0.255 p = 2.75e−07
partial.cor = 0.439 p = 5.88e−20
partial.cor = 0.504 p = 7.34e−27
partial.cor = 0.434 p = 1.72e−19
partial.cor = 0.459 p = 8.52e−22
partial.cor = 0.596 p = 2.16e−39
KICH
cor = 0.144 p = 2.49e−01
partial.cor = 0.479 p = 5.49e−05
partial.cor = 0.333 p = 6.78e−03
partial.cor = −0.076 p = 5.50e−01
partial.cor = 0.345 p = 4.85e−03
partial.cor = 0.026 p = 8.37e−01
partial.cor = 0.399 p = 1.00e−03
KIRC
cor = −0.275 p = 1.93e−09
partial.cor = 0.428 p = 7.43e−22
partial.cor = 0.535 p = 9.40e−34
partial.cor = 0.606 p = 2.06e−47
partial.cor = 0.53 p = 9.54e−34
partial.cor = 0.644 p = 4.19e−55
partial.cor = 0.661 p = 1.90e−58
KIRP
cor = −0.247 p = 5.90e−05
partial.cor = 0.345 p = 1.43e−08
partial.cor = 0.377 p = 3.73e−10
partial.cor = 0.427 p = 6.93e−13
partial.cor = 0.143 p = 2.35e−02
partial.cor = 0.423 p = 1.31e−12
partial.cor = 0.503 p = 7.39e−18
LGG
cor = −0.359 p = 5.51e−16
partial.cor = 0.309 p = 5.00e−12
partial.cor = 0.107 p = 1.90e−02
partial.cor = 0.217 p = 1.70e−06
partial.cor = 0.291 p = 1.11e−10
partial.cor = 0.135 p = 3.09e−03
partial.cor = 0.158 p = 5.49e−04
LIHC
cor = −0.072 p = 1.81e−01
partial.cor = 0.449 p = 1.70e−18
partial.cor = 0.396 p = 2.74e−14
partial.cor = 0.406 p = 4.08e−15
partial.cor = 0.486 p = 1.44e−21
partial.cor = 0.473 p = 1.33e−20
partial.cor = 0.509 p = 8.09e−24
LUAD
cor = −0.354 p = 5.24e−16
partial.cor = 0.412 p = 3.27e−21
partial.cor = 0.305 p = 7.67e−12
partial.cor = 0.607 p = 3.86e−50
partial.cor = 0.319 p = 6.45e−13
partial.cor = 0.548 p = 2.87e−39
partial.cor = 0.564 p = 2.42e−42
LUSC
cor = −0.163 p = 3.57e−04
partial.cor = 0.312 p = 4.43e−12
partial.cor = 0.381 p = 6.91e−18
partial.cor = 0.487 p = 1.04e−29
partial.cor = 0.26 p = 8.20e−09
partial.cor = 0.388 p = 1.53e−18
partial.cor = 0.541 p = 2.27e−37
MESO
cor = −0.353 p = 8.48e−04
partial.cor = 0.183 p = 9.51e−02
partial.cor = 0.035 p = 7.54e−01
partial.cor = 0.28 p = 9.76e−03
partial.cor = 0.106 p = 3.37e−01
partial.cor = 0.205 p = 6.09e−02
partial.cor = 0.353 p = 9.96e−04
FNBP1 Expression Level (log2 TPM)
Infiltration Level

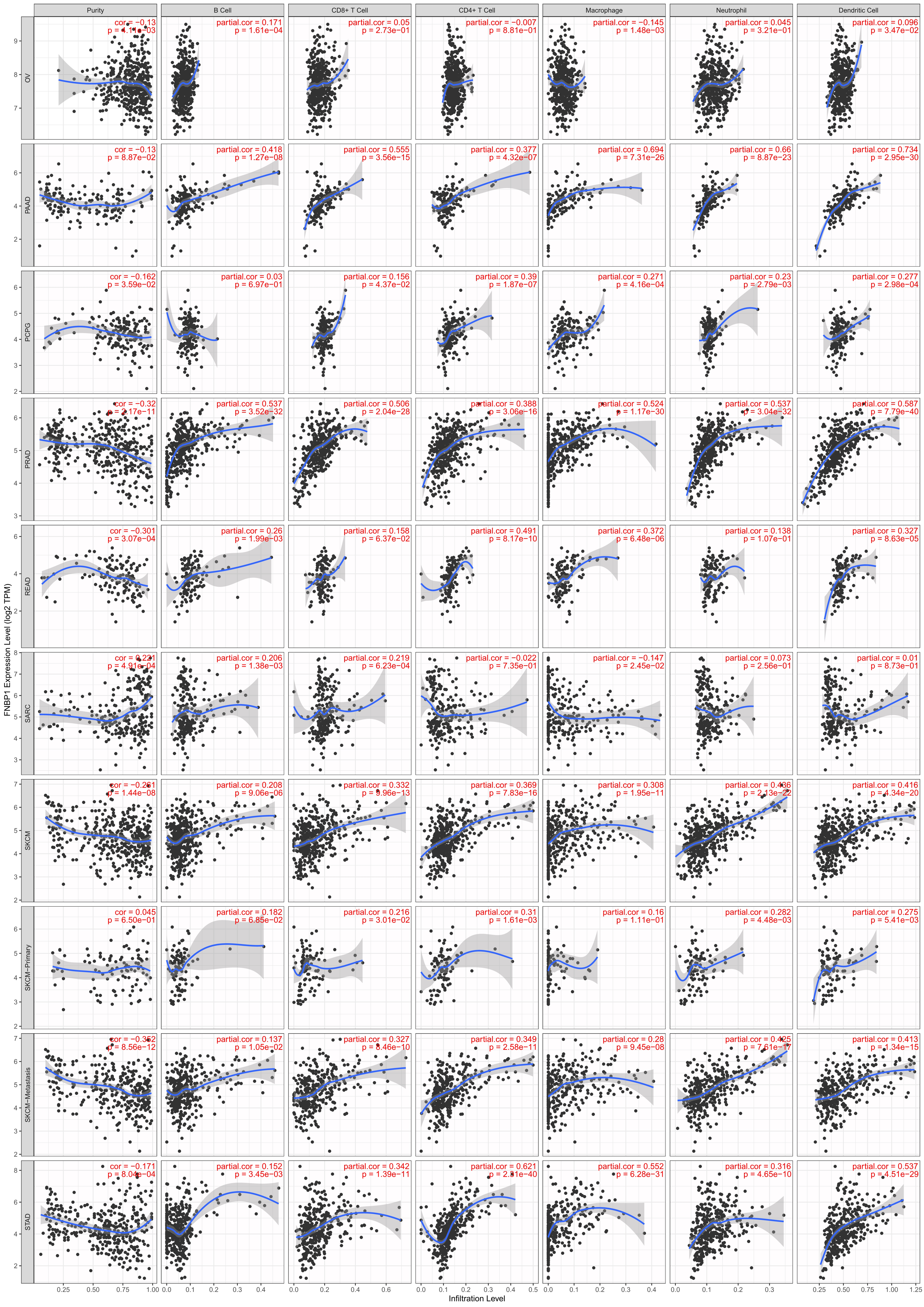
Purity
B Cell
CD8+ T Cell
CD4+ T Cell
Macrophage
Neutrophil
Dendritic Cell
OV
cor = −0.13 p = 4.11e−03
partial.cor = 0.171 p = 1.61e−04
partial.cor = 0.05 p = 2.73e−01
partial.cor = −0.007 p = 8.81e−01
partial.cor = −0.145 p = 1.48e−03
partial.cor = 0.045 p = 3.21e−01
partial.cor = 0.096 p = 3.47e−02
PAAD
cor = −0.13 p = 8.87e−02
partial.cor = 0.418 p = 1.27e−08
partial.cor = 0.555 p = 3.56e−15
partial.cor = 0.377 p = 4.32e−07
partial.cor = 0.694 p = 7.31e−26
partial.cor = 0.66 p = 8.87e−23
partial.cor = 0.734 p = 2.95e−30
PCPG
cor = −0.162 p = 3.59e−02
partial.cor = 0.03 p = 6.97e−01
partial.cor = 0.156 p = 4.37e−02
partial.cor = 0.39 p = 1.87e−07
partial.cor = 0.271 p = 4.16e−04
partial.cor = 0.23 p = 2.79e−03
partial.cor = 0.277 p = 2.98e−04
PRAD
cor = −0.32 p = 2.17e−11
partial.cor = 0.537 p = 3.52e−32
partial.cor = 0.506 p = 2.04e−28
partial.cor = 0.388 p = 3.06e−16
partial.cor = 0.524 p = 1.17e−30
partial.cor = 0.537 p = 3.04e−32
partial.cor = 0.587 p = 7.79e−40
READ
cor = −0.301 p = 3.07e−04
partial.cor = 0.26 p = 1.99e−03
partial.cor = 0.158 p = 6.37e−02
partial.cor = 0.491 p = 8.17e−10
partial.cor = 0.372 p = 6.48e−06
partial.cor = 0.138 p = 1.07e−01
partial.cor = 0.327 p = 8.63e−05
SARC
cor = 0.221 p = 4.91e−04
partial.cor = 0.206 p = 1.38e−03
partial.cor = 0.219 p = 6.23e−04
partial.cor = −0.022 p = 7.35e−01
partial.cor = −0.147 p = 2.45e−02
partial.cor = 0.073 p = 2.56e−01
partial.cor = 0.01 p = 8.73e−01
SKCM
cor = −0.261 p = 1.44e−08
partial.cor = 0.208 p = 9.06e−06
partial.cor = 0.332 p = 9.96e−13
partial.cor = 0.369 p = 7.83e−16
partial.cor = 0.308 p = 1.95e−11
partial.cor = 0.436 p = 2.13e−22
partial.cor = 0.416 p = 4.34e−20
SKCM−Primary
cor = 0.045 p = 6.50e−01
partial.cor = 0.182 p = 6.85e−02
partial.cor = 0.216 p = 3.01e−02
partial.cor = 0.31 p = 1.61e−03
partial.cor = 0.16 p = 1.11e−01
partial.cor = 0.282 p = 4.48e−03
partial.cor = 0.275 p = 5.41e−03
SKCM−Metastasis
cor = −0.352 p = 8.56e−12
partial.cor = 0.137 p = 1.05e−02
partial.cor = 0.327 p = 8.46e−10
partial.cor = 0.349 p = 2.58e−11
partial.cor = 0.28 p = 9.45e−08
partial.cor = 0.425 p = 7.61e−17
partial.cor = 0.413 p = 1.34e−15
STAD
cor = −0.171 p = 8.04e−04
partial.cor = 0.152 p = 3.45e−03
partial.cor = 0.342 p = 1.39e−11
partial.cor = 0.621 p = 2.81e−40
partial.cor = 0.552 p = 6.28e−31
partial.cor = 0.316 p = 4.65e−10
partial.cor = 0.537 p = 4.51e−29
FNBP1 Expression Level (log2 TPM)
Infiltration Level

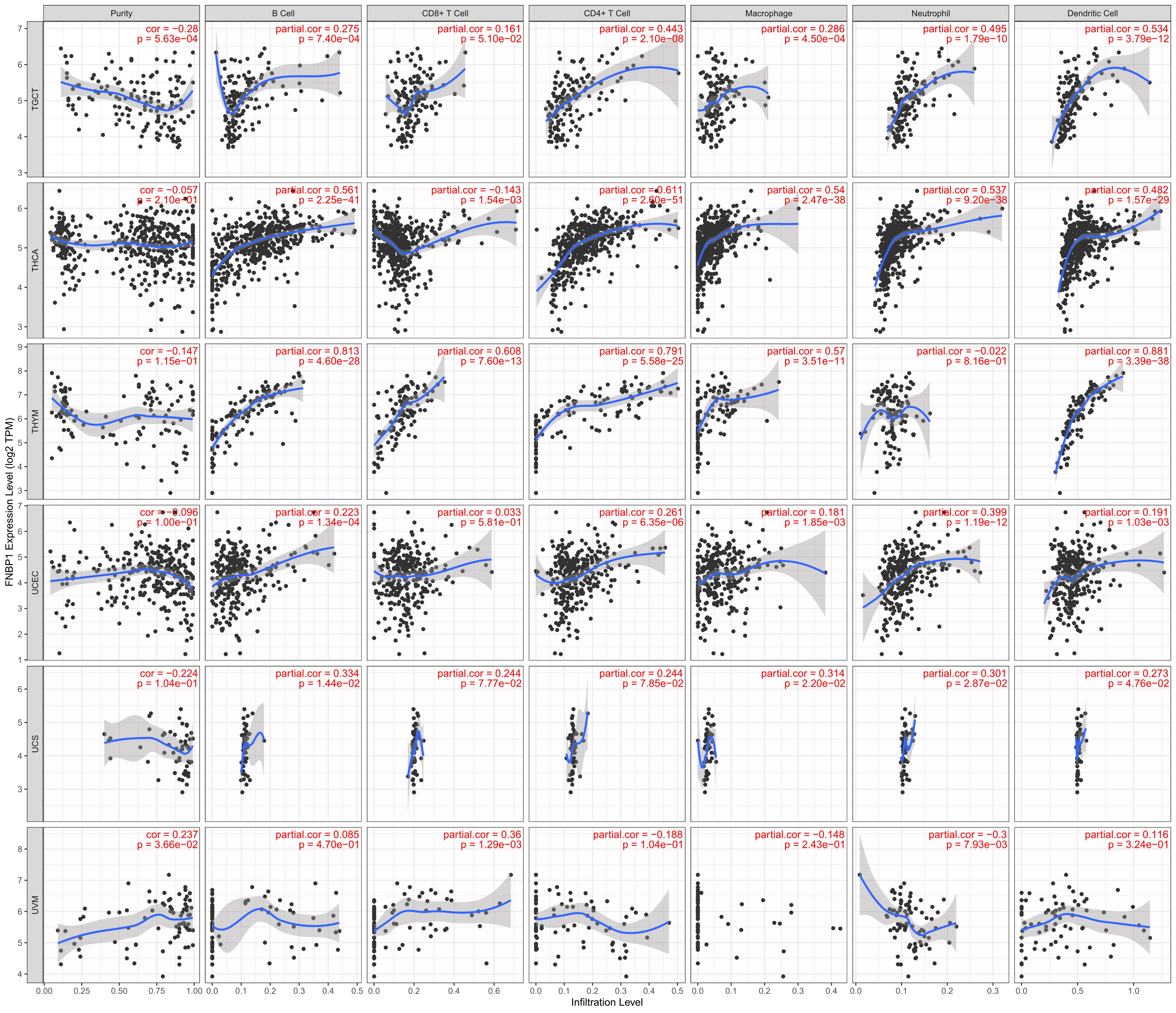
Purity
B Cell
CD8+ T Cell
CD4+ T Cell
Macrophage
Neutrophil
Dendritic Cell
TGCT
cor = −0.28 p = 5.63e−04
partial.cor = 0.275 p = 7.40e−04
partial.cor = 0.161 p = 5.10e−02
partial.cor = 0.443 p = 2.10e−08
partial.cor = 0.286 p = 4.50e−04
partial.cor = 0.495 p = 1.79e−10
partial.cor = 0.534 p = 3.79e−12
THCA
cor = −0.057 p = 2.10e−01
partial.cor = 0.561 p = 2.25e−41
partial.cor = −0.143 p = 1.54e−03
partial.cor = 0.611 p = 2.60e−51
partial.cor = 0.54 p = 2.47e−38
partial.cor = 0.537 p = 9.20e−38
partial.cor = 0.482 p = 1.57e−29
THYM
cor = −0.147 p = 1.15e−01
partial.cor = 0.813 p = 4.60e−28
partial.cor = 0.608 p = 7.60e−13
partial.cor = 0.791 p = 5.58e−25
partial.cor = 0.57 p = 3.51e−11
partial.cor = −0.022 p = 8.16e−01
partial.cor = 0.881 p = 3.39e−38
UCEC
cor = −0.096 p = 1.00e−01
partial.cor = 0.223 p = 1.34e−04
partial.cor = 0.033 p = 5.81e−01
partial.cor = 0.261 p = 6.35e−06
partial.cor = 0.181 p = 1.85e−03
partial.cor = 0.399 p = 1.19e−12
partial.cor = 0.191 p = 1.03e−03
UCS
cor = −0.224 p = 1.04e−01
partial.cor = 0.334 p = 1.44e−02
partial.cor = 0.244 p = 7.77e−02
partial.cor = 0.244 p = 7.85e−02
partial.cor = 0.314 p = 2.20e−02
partial.cor = 0.301 p = 2.87e−02
partial.cor = 0.273 p = 4.76e−02
UVM
cor = 0.237 p = 3.66e−02
partial.cor = 0.085 p = 4.70e−01
partial.cor = 0.36 p = 1.29e−03
partial.cor = −0.188 p = 1.04e−01
partial.cor = −0.148 p = 2.43e−01
partial.cor = −0.3 p = 7.93e−03
partial.cor = 0.116 p = 3.24e−01
FNBP1 Expression Level (log2 TPM)
Infiltration Level
